# Robust and persistent B-cell responses following SARS-CoV-2 vaccine determine protection from SARS-CoV-2 infection

**DOI:** 10.3389/fimmu.2024.1445653

**Published:** 2024-09-17

**Authors:** Joanne Byrne, Lili Gu, Alejandro Garcia-Leon, Colette Marie Gaillard, Gurvin Saini, Dana Alalwan, Julen Tomás-Cortázar, Grace Kenny, Sean Donohue, Bearach Reynolds, Tessa O’Gorman, Alan Landay, Peter Doran, Jannik Stemler, Philipp Koehler, Rebecca Jane Cox, Ole F. Olesen, Jean-Daniel Lelievre, Cathal O’Broin, Stefano Savinelli, Eoin R. Feeney, Jane A. O’Halloran, Aoife Cotter, Mary Horgan, Christine Kelly, Corrina Sadlier, Eoghan de Barra, Oliver A. Cornely, Virginie Gautier, Patrick WG Mallon

**Affiliations:** ^1^ Centre for Experimental Pathogen Host Research (CEPHR), University College Dublin, Dublin, Ireland; ^2^ Department of Infectious Diseases, St Vincent’s University Hospital, Dublin, Ireland; ^3^ Department of Infectious Diseases, Mater Misericordiae University Hospital, Dublin, Ireland; ^4^ Department of Internal Medicine, University of Texas Medical Branch, Galveston, TX, United States; ^5^ School of Medicine, University College Dublin, Dublin, Ireland; ^6^ Faculty of Medicine and University Hospital Cologne, Department I of Internal Medicine, University of Cologne, Cologne, Germany; ^7^ Faculty of Medicine Institute of Translational Research, Cologne Excellence Cluster on Cellular Stress Responses in Aging-Associated Diseases (CECAD), University of Cologne, Cologne, Germany; ^8^ Influenza Centre, Department of Clinical Science, University of Bergen, Bergen, Norway; ^9^ European Vaccine Initiative, Heidelberg, Germany; ^10^ Vaccine Research Institute, Université Paris Est Créteil, Paris, France; ^11^ Department of Infectious Diseases, Cork University Hospital, Cork, Ireland; ^12^ Department of Infectious Diseases, Beaumont Hospital, Dublin, Ireland; ^13^ Department of International Health and Tropical Medicine, Royal College of Surgeons in Ireland, Dublin, Ireland; ^14^ Partner Site Bonn-Cologne Department Cologne, German Centre for Infection Research (DZIF), Cologne, Germany

**Keywords:** SARS-CoV-2, COVID-19, COVID-19 vaccine, immunogenicity, B cells, T cells

## Abstract

**Introduction:**

A clear immune correlate of protection from severe acute respiratory syndrome coronavirus 2 (SARS-CoV-2) infection has not been defined. We explored antibody, B-cell, and T-cell responses to the third-dose vaccine and relationship to incident SARS-CoV-2 infection.

**Methods:**

Adults in a prospective cohort provided blood samples at day 0, day 14, and 10 months after the third-dose SARS-CoV-2 vaccine. Participants self-reported incident SARS-CoV-2 infection. Plasma anti–SARS-CoV-2 receptor-binding domain (RBD) and spike-subunit-1 and spike-subunit-2 antibodies were measured. A sub-study assessed SARS-CoV-2–specific plasma and memory B-cell and memory T-cell responses in peripheral blood mononuclear cells by enzyme-linked immunospot. Comparative analysis between participants who developed incident infection and uninfected participants utilised non-parametric t-tests, Kaplan–Meier survival analysis, and Cox proportional hazard ratios.

**Results:**

Of the 132 participants, 47 (36%) reported incident SARS-CoV-2 infection at a median 16.5 (16.25–21) weeks after the third-dose vaccination. RBD titres and B-cell responses, but not T-cell responses, increased after the third-dose vaccine. Whereas no significant difference in day 14 antibody titres or T-cell responses was observed between participants with and without incident SARS-CoV-2 infection, RBD memory B-cell frequencies were significantly higher in those who did not develop infection [10.0% (4.5%–16.0%) versus 4.9% (1.6%–9.3%), p = 0.01]. RBD titres and memory B-cell frequencies remained significantly higher at 10 months than day 0 levels (p < 0.01).

**Discussion:**

Robust antibody and B-cell responses persisted at 10 months following the third-dose vaccination. Higher memory B-cell frequencies, rather than antibody titres or T-cell responses, predicted protection from subsequent infection, identifying memory B cells as a correlate of protection.

## Introduction

Severe acute respiratory syndrome coronavirus 2 (SARS-CoV-2) vaccination markedly decreases the risk of progression to severe coronavirus disease 2019 (COVID-19) and death (1). However, breakthrough infections occur despite repeated vaccinations. Although both waning immunity and immune escape driven by viral evolution are thought to contribute to breakthrough infections, a clear immune correlate of protection from infection has not been defined and questions remain about which element of the immune response to vaccination best predicts long-term immunity.

Memory B cells play a crucial role in the immune system’s anamnestic ability to mount a rapid and enhanced response upon pathogen re-exposure, undergoing clonal expansion and differentiation into antigen-secreting cells. The two-dose mRNA-based vaccination has been shown to effectively induce a memory B-cell response (2), which is enhanced by the third-dose vaccine (3). Although neutralising antibodies declined over time following vaccination, spike (S)–specific and RBD-specific memory B-cell responses remained detectable at 6 months after primary vaccination (3). Specialised compartments, such as the germinal centre (GC) in lymph nodes, house distinct subsets of B cells where B cells acquire affinity-enhancing somatic hypermutations (SHMs). Notably, high sustained frequencies of S-binding GC B cells and plasma cells were demonstrated in draining lymph nodes for at least 12 weeks after the third-dose vaccination facilitating the generation of robust humoral immunity to SARS-CoV-2 (4). Examination of the GC in immunocompromised subjects has also demonstrated blunted SARS-CoV-2–specific GC B-cell responses and reduced SARS-CoV-2 RBD-specific memory B cells than healthy subjects, which may explain the reduced neutralising antibody responses seen in immunocompromised individuals again underscoring the critical role of B-cell GCs in neutralising antibody development (5). One cohort study demonstrated that individuals experiencing vaccine breakthrough infection exhibited reduced frequencies of SARS-CoV-2 receptor-binding domain (RBD)–specific memory B cells at diagnosis in contrast to their vaccinated close contacts who did not develop infection (6). However, no prospective studies have explored the relative contribution of memory B-cell or plasma cell responses as compared to circulating binding antibody titres or T-cell responses after vaccination as a reliable predictor of future protection from infection.

Neutralisation capacity is the gold-standard surrogate of underlying host immunity. Cohort studies analyses and aggregated clinical trial data have reported correlations between circulating binding antibody concentrations and both underlying host viral neutralising capacity and protection from COVID-19 (7–11). The emergence of variants of concern (VOCs) further complicates the search for a correlate of protection against SARS-CoV-2 infection. The Omicron (OM; B.1.1.529) VOC showed greater escape from vaccine-elicited neutralising antibody responses in comparison to wild-type (WT) virus (12, 13). Although an absolute circulating antibody level as a correlate of protection has been the subject of much research (14), a recent study determined an anti-RBD immunoglobulin G (IgG) threshold of 456 binding antibody units (BAU)/ml to correspond to a clinically relevant underlying host neutralising capacity against both WT and immune escape variants of SARS-CoV-2 including OM (15).

SARS-CoV-2 primary vaccination leads to induction of SARS-CoV-2–specific T-cell responses (16). However, booster doses of mRNA vaccines have been shown to have little effect on S-specific CD8+ T memory stem pool memory frequencies, which remained constant after three and four vaccine booster doses (17). Memory T cells, rather than memory B cells, have been demonstrated as the rapid responders during breakthrough SARS-CoV-2 infection enhancing the adaptive immune response (18). Yet, conflicting data exist regarding the role of SARS-CoV-2–specific T-cell responses in protection against infection. Whereas one cohort study observed higher SARS-CoV-2–specific memory T cells in close contacts at the time of exposure compared to diagnosed cases (19), another study found no significant difference in T-cell responses between infected individuals and their negative close contacts at the time of infection (6). A definitive role for vaccine-elicited memory T-cell responses as a correlate of protection from SARS-CoV-2 infection remains uncertain.

Comprehensive, prospective, multi-parameter assessments of these immune factors after booster vaccination, alongside their protective efficacy against future infection, have not yet been conducted in a real-world setting. We aimed to explore SARS-CoV-2–specific B-cell responses, circulating antibody levels, and T-cell responses before and after the third-dose vaccine and determine which element of the immune response predicted protection from subsequent SARS-CoV-2 infection.

## Methods

### Study design and participants

The All-Ireland Infectious Diseases (AIID) Cohort Study is a prospective, multicentre, observational study recruiting individuals with issues pertaining to infectious diseases (approved by the National Research Ethics Committee in Ireland, reference 20-NREC-COV-056). Adult (≥18 years) participants provided a written informed consent in accordance with the Declaration of Helsinki for collection of clinical data and biobanking of bloods including ethylenediamine tetraacetic acid–derived plasma (stored at −80°C) and cryopreserved peripheral blood mononuclear cells (PBMCs) (in liquid nitrogen). For this analysis, all AIID cohort participants receiving the third dose of SARS-CoV-2 vaccine in November 2021 and with blood samples available at day 0, day 14, and 10 months were included. All included participants were healthcare workers and received the monovalent Pfizer-BioNTech BNT162b2 as was national first-line policy at the time. Data on self-reported SARS-CoV-2 incident infection (PCR or antigen test confirmed) was collected during the two follow-up periods. Given the changing nature of circulating SARS-CoV-2 variants, participants were assumed to have contracted the Delta (B.1.617.2) variant if reporting a positive test before 12 December 2021 and OM (B.1.1.529) after this date (20).

### Measurement of immune responses

Circulating antibody titres were evaluated on samples at the day 0 and day 14 time points for all participants. B-cell responses were analysed at day 0 and day 14 after the third-dose vaccination in a sub-study where participants were matched based on age and gender. An additional analysis of T-cell, B-cell, and circulating antibody responses was also performed in samples from a subset of participants with samples available at 10 months after the third-dose vaccination. Notably, all of participants in this sub-analysis were also included in the B-cell analysis.

We used a quantitative electrochemiluminescence assay to quantify antibodies to WT SARS-CoV-2 spike subunit 1 (S1), spike subunit 2 (S2), and RBD in plasma using the Centre for Experimental Pathogen Host Research (CEPHR) COVID-19 serologic assay, described in detail elsewhere (21), with results reported in World Health Organisation (WHO)–standardised BAU/mL.

We assessed SARS-CoV-2–specific plasma cell and memory B-cell responses from PBMCs. Plates were coated with SARS-CoV-2 WT RBD and full-S antigens (Sino Biological Inc., China) or anti-human IgG (Mabtech, Sweden) serving as controls. For the measurement of memory B-cell frequencies, cells were stimulated *ex vivo* with R848 and interleukin-2 (IL-2) to differentiate into antibody-secreting cells (ASCs) and analysed by enzyme-linked immunospot (ELISpot) Mabtech, Sweden). For the plasma cells, antigen-specific IgG-secreting B cells were expressed as spot-forming unit (SFU) per 10^6^ PBMCs. For the memory B cells, antigen-specific IgG-secreting B cells were expressed as SFU per total IgG-secreting B cells (IgG+ ASC) controls.

We assessed SARS-CoV-2–specific memory T-cell responses from PBMCs after stimulation, with SARS-CoV-2 WT S and nucleocapsid (N) peptides and OM BA4&BA5 S and N peptides (Sino Biological Inc., China) by ELISpot. The SARS-CoV-2–specific memory T cells were expressed as SFU per 0.5 × 10^6^ PBMCs subtracted with negative control. Detailed methods are provided in the **Supplementary Materials**.

### Statistical analysis

Continuous variables were summarised using median and interquartile range (IQR) and categorical variables with frequency and percent. We used Wilcoxon signed-rank test and Mann–Whitney U test for paired and unpaired comparisons, respectively. Kaplan–Meier survival analysis and Cox proportional hazard regression analysis were used to explore relationships between variables of interest and incident infection, adjusting for age, sex, and antibody titres. Statistical analysis was performed using R (version 4.3.1) software and Prism version 10.0.2 (GraphPad).

## Results

### Study population

**Figure 1:**
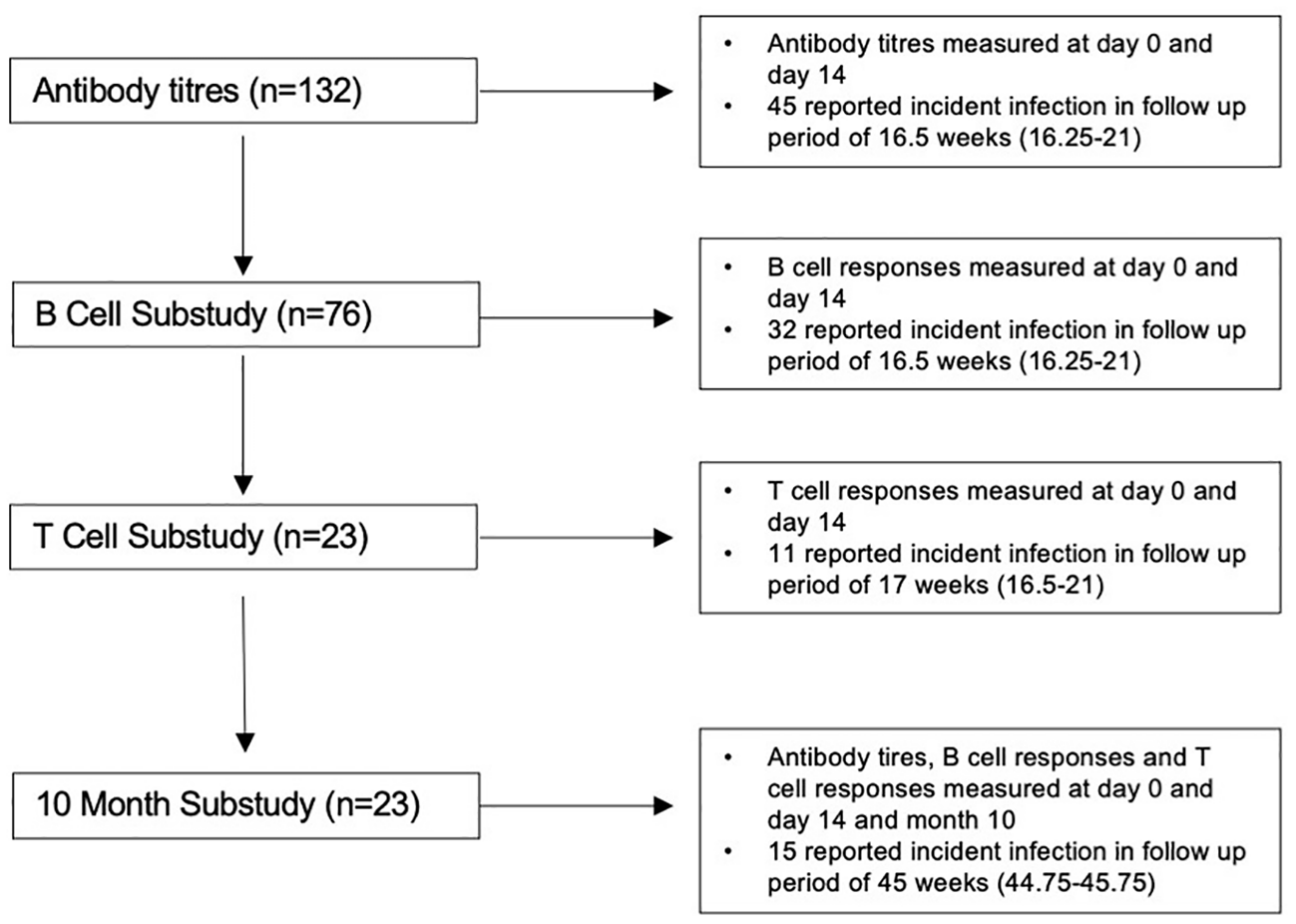
Flow diagram of study population.

Of the 132 participants recruited to the analysis, 76 were included in the B-cell sub-analysis and 23 contributed data to the T-cell sub-analysis and 10 month follow-up sub-analysis (summarised in **Figure 1**). For all included participants, the median (IQR) age was 43 (32–50), 81% were women, and 101 (77%) had received primary two-dose vaccination with Pfizer-BioNTech BNT162b2 (demographics are summarised in **Table 1**). The median follow-up for all included participants was 16.5 (16.25–21) weeks follow-up. Forty-seven (36%) reported incident SARS-CoV-2 infection, at a median interval of 90 days (57–112.5) after the third-dose vaccination. Forty (85%) were symptomatic, and 46 (98%) were presumed to have been infected with an OM variant due to date of infection (20). For the 23 participants with data available at 10 months [median, 45 weeks (44.75–45.75)], 15 (65%) reported incident infection at a median 71 days (53–141) after the third-dose vaccination, an additional four participants subsequent to the first follow-up period. All participants had mild disease as classified by the WHO (22).

**Table 1:** Characteristics of the study population.

	Full study cohort		B-cell sub-analysis		T-cell sub-analysis	
	Infection* (n = 47)	No infection (n = 85)	Infection* (n = 32)	No infection (n = 46)	Infection* (n = 11)	No infection (n = 12)
*Age (years)*	41 (33–47.5)	43 (32–51)	40 (34.5–47)	39 (29.5–51.5)	43 (37–51)	38 (31–48.5)
*Female sex [n (%)]*	33 (70%)	74 (87%)	20 (63%)	36 (82%)	5 (45%)	9 (75%)
*Previous (before booster)* *SARS-CoV-2 infection [n (%)]*	7 (15%)	14 (16%)	7 (22%)	5 (11%)	1	0
*Months since previous infection* *[median (IQR)]*	10 (8.5–17)	10 (8.5–17)	10 (8.5–17)	10 (10–19)	3	NA
*Initial vaccinations [n (%)]* *Pfizer-BioNTech BNT162b2* *AstraZeneca ChAdOx1 nCoV-19*	38 (81%) 9 (19%)	63 (74%) 22 (26%)	27(84%) 5(16%)	33(75%) 11(25%)	8 (73%) 3 (27%)	10 (83%) 2 (17%)
*Days since the second vaccine*	283 (245–286)	282 (188–286)	284 (252–286)	284 (193–286)	285 (225–286)	285 (261–289)
*Symptomatic infection*	40 (85%)	NA	26 (81%)	NA	6 (55%)	NA

*Infection as self-reported by participants by SARS-CoV-2 PCR or antigen test during follow-up period [median (IQR)] of 16.5 (16.25–21) weeks.

### Circulating antibody response to the third-dose SARS-CoV-2 vaccine

**Figure 2:**
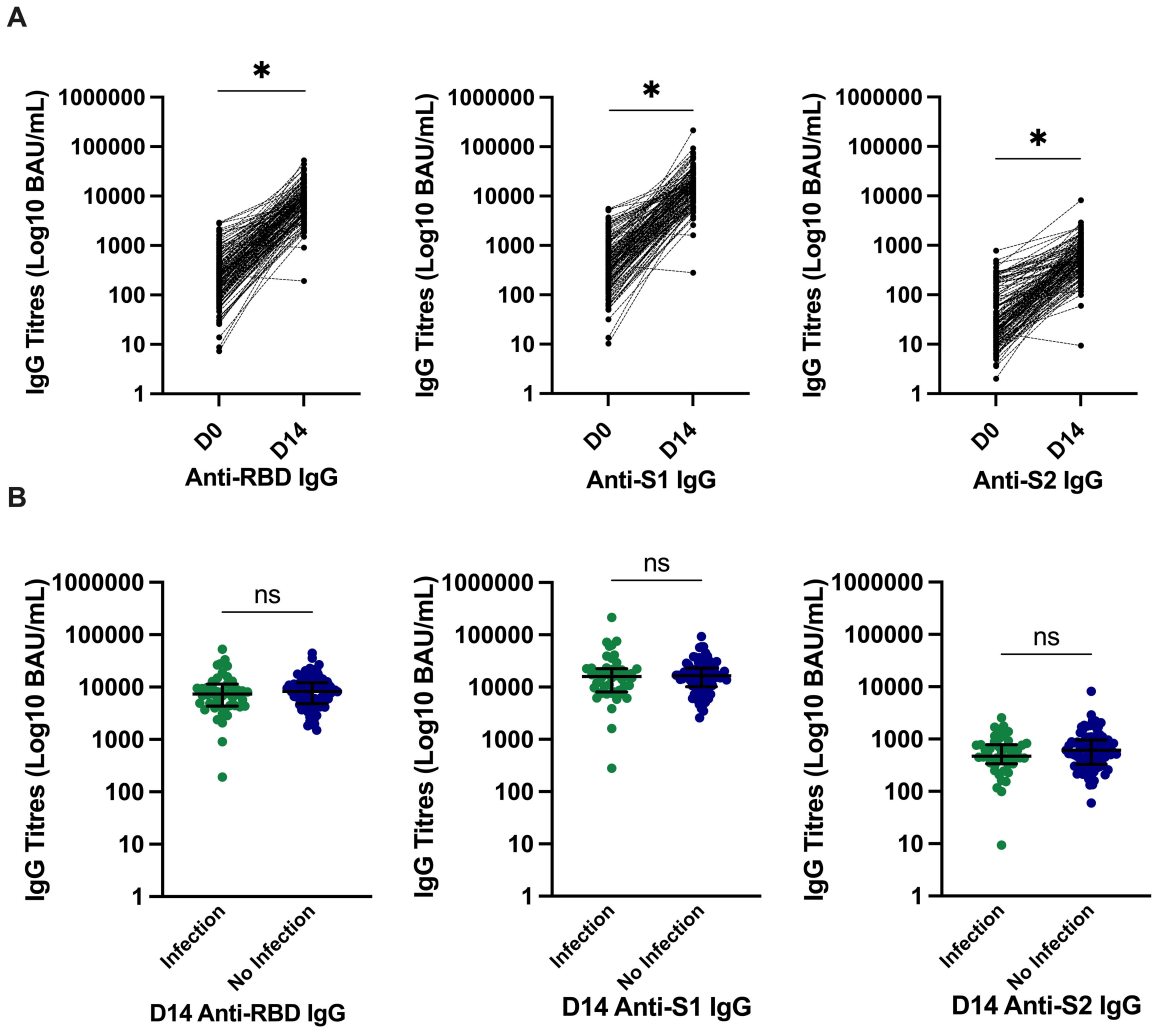
SARS-CoV-2–specific antibody titres and relationship with incident infection. **(A)** Significantly higher SARS-CoV-2–specific anti-RBD, anti-S1, and anti-S2 titres at day 14. *p ≤ 0.0001 compared to pre-vaccine response by Wilcoxon signed-rank test. **(B)** There was no significant difference in anti-RBD IgG, anti–spike-subunit-1 IgG, or anti–spike-subunit-2 IgG titres at day 14 in those who developed infection and those who did not develop infection. Bars represent median and interquartile range. *P-*values calculated by Mann–Whitney U test are shown above each comparison. D0, day 0; D14, day 14; RBD, receptor-binding domain; S, spike; S1, spike subunit 1; S2, spike subunit 2; BAU, binding antibody unit; IgG Immunoglobulin G.

The median (IQR) circulating antibody titres (BAU/mL) increased significantly from day 0 to day 14 from 211 (103–414) to 8,162 (4,581–11,914) for anti-RBD, 386 (196–728) to 16,305 (9,585–22,623) for anti-S1, and 24 (14–79) to 570 (335–888) for anti-S2; all *p* < 0.0001 (**Figure 2A**). There was no significant difference in day 14 circulating antibody titres (BAU/mL) between participants with and without subsequent infection. The median (IQR) of those who developed infection were [anti-RBD, 7,332 (4404–11017); anti-S1, 16,011 (8,405–22,427); and anti-S2, 470 (345–773)] versus those who did not develop infection [anti-RBD, 8,310 (4,896–11,959); anti-S1, 16,449 (10,659–22,993); and anti-S2, 612 (335–955)]; all *p* > 0.05 (**Figure 2B**).

### B-cell response to the third-dose SARS-CoV-2 vaccination

**Figure 3:**
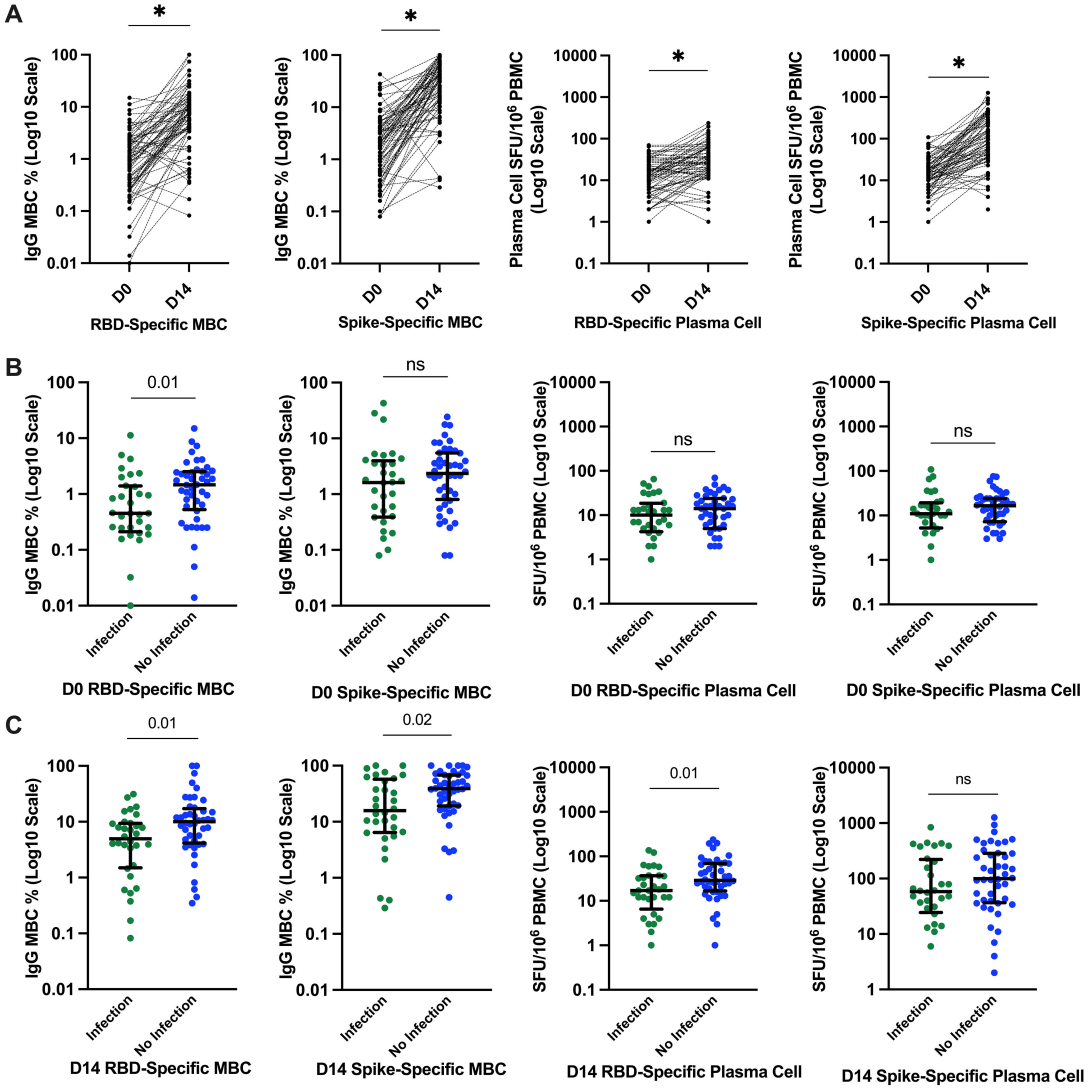
SARs-CoV-2–specific B-cell responses and relationship with incident infection. **(A)** Significantly higher SARS-CoV-2–specific memory B-cell and plasma cell responses at day 14. *p ≤ 0.0001 compared to pre-vaccine response by Wilcoxon signed-rank test. **(B)** SARS-CoV-2–specific RBD and S plasma cell and memory B-cell responses before the third-dose SARS-CoV-2 vaccination and relationship with incident SARS-CoV-2 infection. Bars represent median and interquartile range. *P-*value calculated by Mann–Whitney U test are shown above each comparison. **(C)** SARS-CoV-2–specific RBD and S plasma cell and memory B-cell responses 14 days after the third-dose SARS-CoV-2 vaccination and relationship with incident SARS-CoV-2 infection. D0, day 0; D14, day 14; PBMCs, peripheral blood mononuclear cells; RBD, receptor-binding domain; S, spike; ns, not significant; SFU, spot-forming unit.

We evaluated B-cell function through SARS-CoV-2 RBD and full-S–specific memory B and plasma cell responses. The median (IQR) RBD and S-specific memory B cells (% total IgG-secreting B cells) increased significantly from day 0 to day 14, from 0.95 (0.26–2.26) to 7.86 (3.83–13.55) for RBD-specific and 2.5 (0.58–4.2) to 33.62 (12.63–63.48) for S-specific. Similarly, the median (IQR) plasma cell responses (SFU/10^6^ PBMCs) increased significantly from day 0 to day 14, from 12 (5–21.5) to 25.5 (12–58.5) for RBD-specific and 14 (7–24) to 79.5 (35.5–268.5) for S-specific; all *p* < 0.0001 (**Figure 3A**). There was no significant difference in memory B-cell frequency–based. Linear regression analysis of the day 14 S-specific memory B-cell responses indicated that the vaccine type used for priming did not significantly impact the day 14 responses (p = 0.95), suggesting that the observed increase in B-cell responses is consistent across different vaccine types.

In contrast to what we observed with circulating antibody responses, those reporting incident SARS-CoV-2 infection had significantly lower memory RBD-specific B-cell frequencies both at day 0 and day 14 compared to those who did not report incident SARS-CoV-2 infection. The median (IQR) RBD memory B-cell frequencies (% total IgG-secreting B cells) at day 0 were 0.45 (0.24–1.36) versus 1.46 (0.58–2.48) (**Figure 3B**) and the median day 14 RBD-specific memory B-cell frequencies 4.94 (1.6–9.26) versus 10.03 (4.45–16) in those with and without incident SARS-CoV-2 infection, respectively (**Figure 3C**). Similarly, day 14 full-S–specific memory B-cell frequencies were significantly lower in those with incident SARS-CoV-2 infection with median of 15.75 (6.52–54.4) versus 38.94 (19.34–67.24) in those without incident SARS-CoV-2 infection (**Figure 3C**).

Day 14 RBD-specific plasma cell responses (SFU/10^6^ PBMCs) were also significantly lower in those with incident infection with median of 17 (9.5–36.25) versus 29 (18.25–69) in those without incident SARS-CoV-2 infection (**Figure 3C**). However, day 14 S-specific plasma cell responses were not significantly different between the groups 58 (27.5–208.5) versus 99.5 (38.25–283.25) in those with and without incident infection, respectively, *p* = 0.15 (**Figure 3C**).

### Memory T-cell response to the third-dose SARS-CoV-2 vaccine

**Figure 4:**
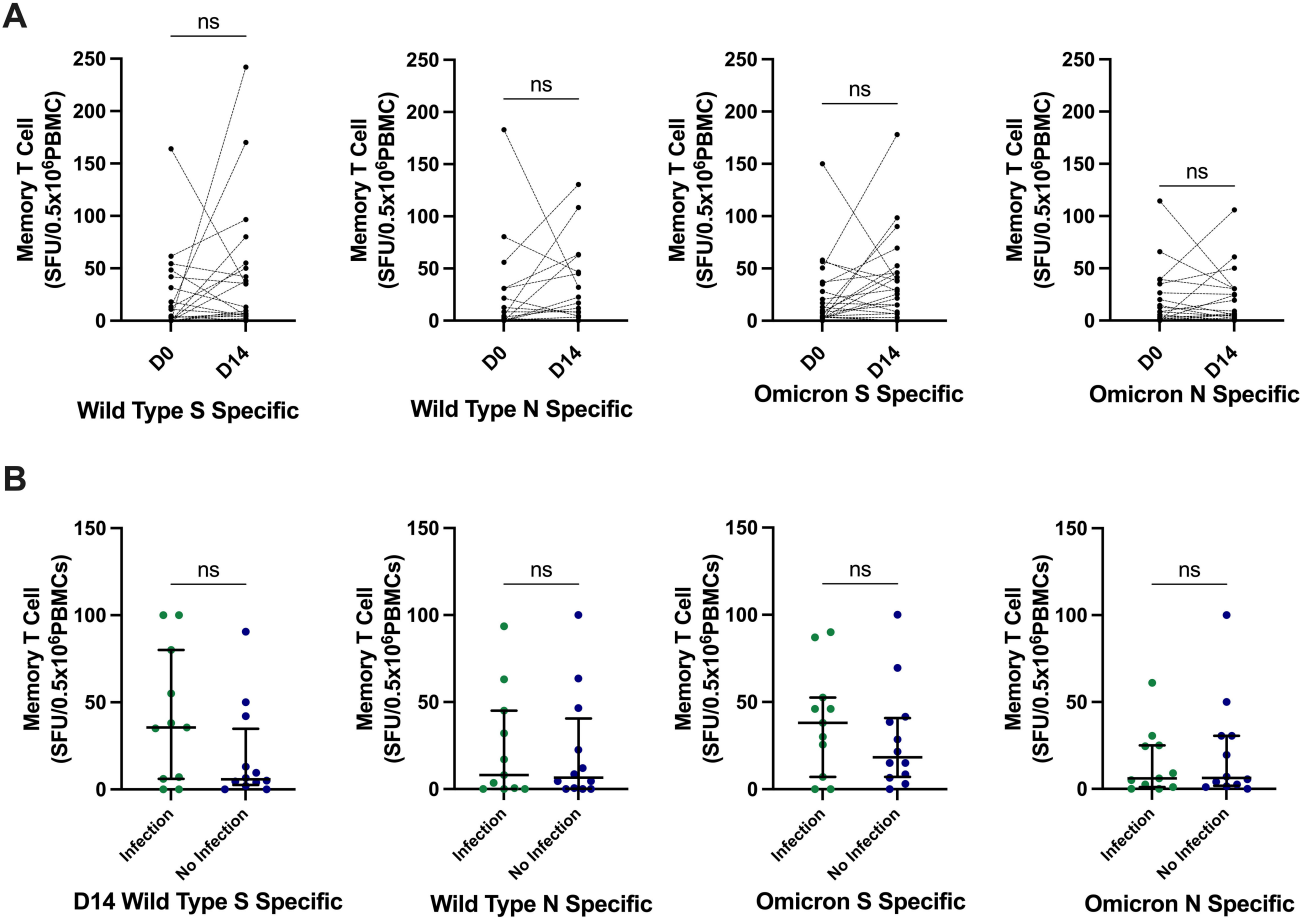
SARS-CoV-2–specific memory T-cell responses and relationship with incident infection. **(A)** There was no significant difference in SARS-CoV-2–specific memory T-cell responses at day 14. *P-*values calculated by Wilcoxon signed-rank test compared to pre-vaccine responses. **(B)** There was no significant differences in the day 14 memory T-cell responses between those who developed infection and those who did not develop infection. *P-*values calculated by Mann–Whitney U test. Bars represent median and interquartile range. S, spike; N, nucleocapsid; SFU, spot-forming unit; PBMCs, peripheral blood mononuclear cells; ns, not significant.

We investigated T-cell function through full-S– and N-specific memory T-cell responses directed against WT and OM variants in a subset (N = 23) of participants at day 0 and day 14 after the third-dose vaccination, of whom 11 (45%) reported incident SARS-CoV-2 infection during the initial follow-up period of median (IQR) 17 weeks (16.5–21). WT T-cell responses (SFU/0.5 × 10^6^ PBMCs) did not significantly change from day 0 to day 14, from median of 9.5 (4.25–46) to 3.5 (0–24.75) for WT S and of 8 (0.25–38.5) to 2 (0–21.5) for WT N. Similarly, OM-specific T-cell responses did not significantly change from day 0 to day 14, from median of 28.5 (7.75–46) to 8.5 (3.25–31.5) for OM S and of 6 (2–27.75) to 5.5 (0–23.25) for OM N; all *p* > 0.05 (**Figure 4A**). There was also no significant difference in T-cell responses between those who developed infection and those who did not (**Figure 4B**).

### Immune responses at 10 months after the third-dose SARS-CoV-2 vaccine

**Figure 5:**
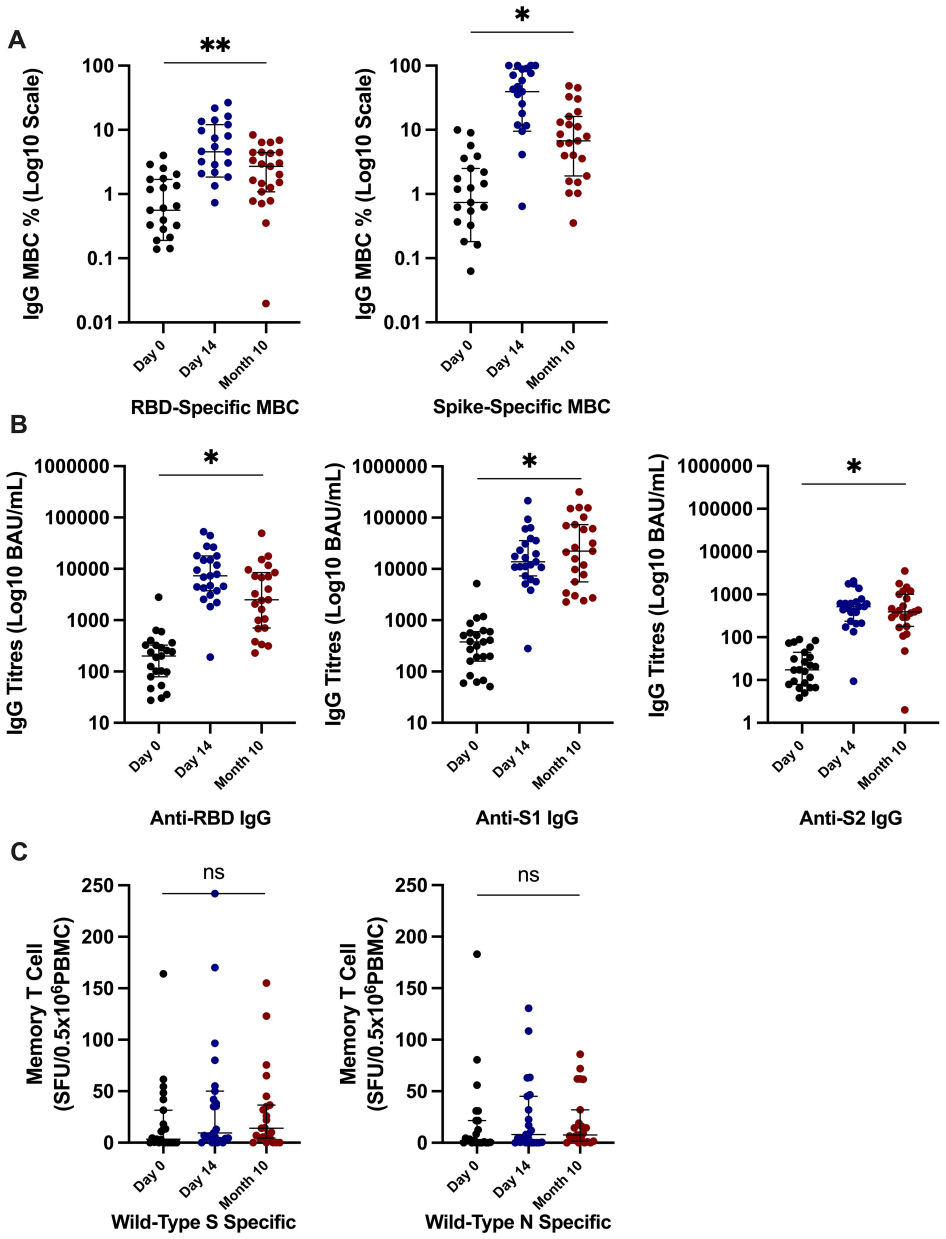
Immune responses at 10 months after the third-dose vaccine. **(A)** Significantly higher SARS-CoV-2–specific memory B-cell frequencies at 10 months after third-dose vaccine in comparison to day 0 frequencies. **(B)** Significantly higher anti-RBD, anti-S1, and anti-S2 titres at 10 months after the third-dose vaccine in comparison to day 0 responses. **(C)** There was no significant difference in SARS-CoV-2–specific memory T-cell responses at month 10 in comparison to day 0 responses. *P-*value calculated by Wilcoxon signed-rank of responses at 10 months in comparison to day 0 responses. **p = 0.01, *p ≤ 0.0001; ns, not significant. RBD, receptor-binding domain; S, full spike; S1, spike subunit 1; S2, spike subunit 2; N, nucleocapsid; WT, wild type; MBC, memory B cell; PBMCs, peripheral blood mononuclear cells, BAU, binding antibody unit; SFU, spot-forming unit.

We evaluated antibody, B-cell responses, and T-cell responses in the same subset (N = 23) of participants additionally at 10 months after the third-dose vaccine; 15 (65%) self-reported SARS-CoV-2 infection at 10 months after the third-dose vaccine. We observed robust, persistent memory B-cell frequencies, with levels maintained significantly above those recorded at day 0. RBD-specific memory B cells (% total IgG-secreting B cells) were median of 2.71 (1.17–4.46) at month 10 versus 0.95 (0.31–2.11) at day 0 (*p* = 0.01). S-specific memory B cells (% total IgG-secreting B cells) were median of 6.74 (2.73–14.66) at month 10 versus 2.2 (0.96–4.05) at day 0 (*p* = 0.01) (**Figure 5A**). Circulating antibody titres (BAU/mL) were also significantly higher at month 10 after vaccine compared to day 0 (anti-RBD titres median of 2,498 (850–7840) versus 200 (28–326), anti-S1 titres of 22,188 (2,267–71,619) versus 378 (175–581), and anti-S2 titres of 365 (204–608) versus 17 (8–39) at month 10 versus day 0, respectively; all *p* < 0.0001 (**Figure 5B**). Nineteen (83%) participants had anti-RBD titres >456 BAU/m, versus 3 (13%) participants at day 0, a threshold that has previously been demonstrated to predict clinically relevant host neutralising capacity against SARS-CoV-2 (15). There was no significant differences in memory T-cell responses (SFU/0.5 × 10^6^ PBMCs) at month 10 from day 0 [WT RBD-specific at 14.00 (5–35.75) versus 3.5 (0–24.75) SFU and WT N-specific at 7.5 (2–25.5) versus 2.0 (0–21.5) at month 10 versus day 0 respectively; **Figure 5C**].

### Impact of heterologous prime-boost vaccination on memory B-cell responses

We investigated the impact of heterologous prime-boost vaccination on memory B-cell responses at day 14 after booster vaccination. There was no significant difference in day 14 RBD-specific memory B-cell frequencies between those primed with Pfizer-BioNTech (n = 60) with a median (IQR) of 7.86 (3.85–13.6) and those who had a heterologous prime-boost vaccination with AstraZeneca (n = 16) who had a median (IQR) of 7.32 (3.45–13.7); *p* = 0.99. Similarly, there was no significant difference in S-specific memory B-cell frequencies at day 14 after the third-dose vaccination between those primed with Pfizer-BioNTech [28.3 (11.6–63.9)] and those primed with AstraZeneca [46.4 (16.4–60.6)]; *p* = 0.41.

### Higher memory B-cell and plasma cell responses independently predict protection from infection

**Figure 6:**
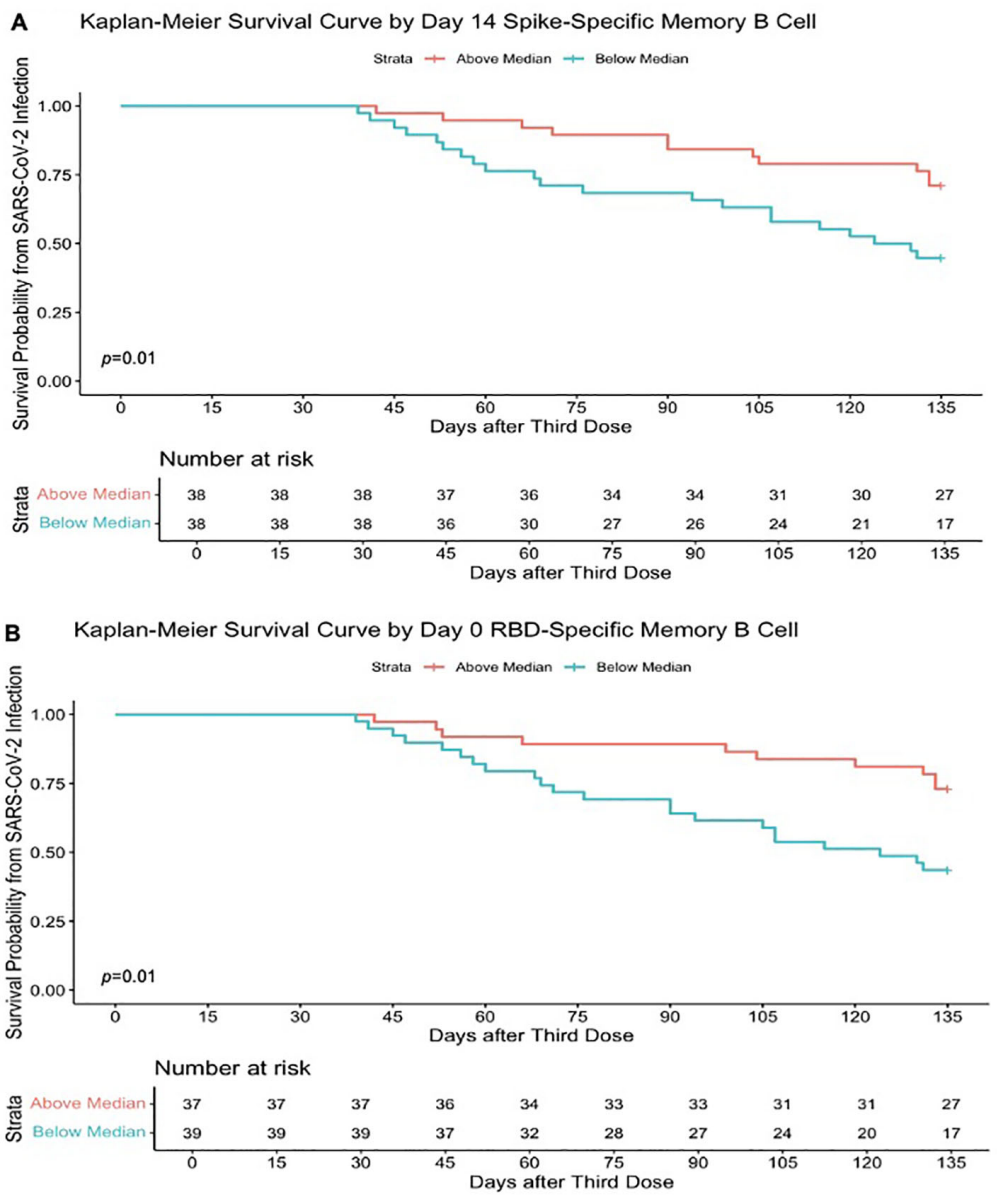
Higher SARS-CoV-2–specific memory B-cell frequencies determine survival from SARS-CoV-2 infection. **(A)** Kaplan–Meier survival curve based on the median SARS-CoV-2 full spike–specific memory B-cell frequencies (33.62% total IgG-secreting B cells) at day 14 after the third-dose SARS-CoV-2 vaccination and incident infection. **(B)** Kaplan–Meier survival curve based on the median SARS-CoV-2 RBD memory B-cell frequencies (0.95% total IgG-secreting B cells) at day 0 after SARS-CoV-2 vaccination and incident infection. Survival analysis *p-*value calculated by log-rank (Mantel–Cox) test.

**Figure 7:**
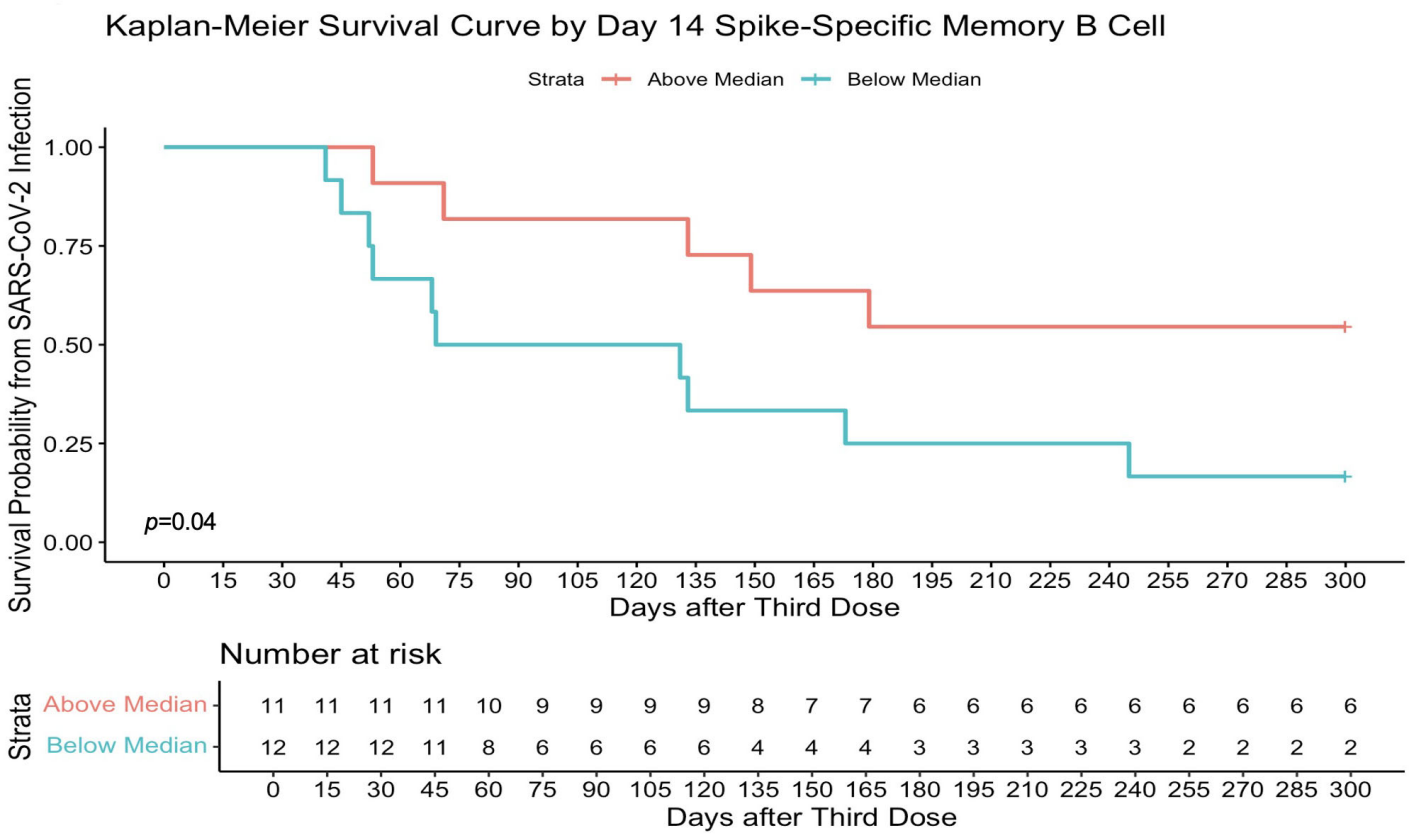
Higher spike-specific memory B-cell frequencies determine survival from SARS-CoV-2 infection at 10 months. Day 14 full spike–specific memory B-cell responses above the median (36.80% total IgG-secreting B cells) were significantly associated with protection from infection in a survival analysis at 10 months after the third-dose vaccination. Survival analysis *p-*value calculated by log-rank (Mantel–Cox) test.

Higher memory B-cell and plasma cell responses at day 14 after the third-dose vaccine were independently associated with protection from incident SARS-CoV-2 infection during the follow-up period of 16.5 (16.25–21) weeks. On univariate analysis, higher day 14 S-specific memory cell frequencies above the median (33.62% total IgG-secreting B cells) were associated with a 59% reduction in risk of incident infection [HR, 0.41 (95% CI, 0.20–0.85); *p* = 0.02; **Figure 6A**]. This strengthened when adjusted for age, sex, and day 14 anti S-1 titres [aHR, 0.33 (95% CI, 0.16–0.69); *p* = 0.01]. Day 14 RBD-specific memory B-cell frequencies above the median (7.86% total IgG-secreting B cells) were also associated with a 54% reduction in risk of incident infection [HR, 0.46 (95% CI, 0.22–0.98); *p* = 0.04]. Higher day 0 RBD-specific memory B-cell frequencies were also associated with protection from incident SARS-CoV-2 infection, with day 0 RBD-specific memory B frequencies above the median (0.95% total IgG-secreting B cells) associated with a 63% reduction in risk of incident infection at 16.5 (16.25–21) weeks [HR, 0.37 (95% CI, 0.17–0.78); *p* = 0.01], when adjusted for age, sex, and day 0 RBD titres [aHR, 0.36 (95% CI, 0.16–0.80); *p* = 0.01] (**Figure 6B**). Similarly, day 14 S-specific plasma cell responses above the median (79.5 SFU/10^6^ PBMCs) also were associated with a 75% reduction in risk of incident infection [aHR, 0.25 (95% CI, 0.12–0.53); *p* = 0.01] and day 14 RBD-specific plasma cell responses above the median (25.5 SFU/10^6^ PBMCs) with a 65% reduction in risk of incident infection [aHR, 0.35 (95% CI, 0.15–0.82); *p* = 0.02] when adjusted for age, sex, and day 14 anti-S1 or anti-RBD titres, respectively. This protective effect was not observed with other B-cell responses before the third-dose vaccine. In addition, the median day 14 antibody titres or T-cell responses after the third-dose vaccination were not associated with a reduction in risk of incident infection. In the 10-month sub-analysis, the protective effect of higher day 14 S-specific memory B-cell frequencies was seen up to 10 months, where responses above the median (36.8% total IgG-secreting B cells) in this sub-analysis were associated with 65% reduction in risk of incident infection at 10 months after vaccination [HR, 0.45 (95% CI, 0.11–0.98); *p* = 0.05; **Figure 7**] and strengthened when adjusted for age, sex, and day 14 S-specific titres [aHR, 0.27 (95% CI, 0.07–0.95); *p* = 0.04].

## Discussion

This is the first study evaluating a multi-parameter immune response to the third-dose SARS-CoV-2 vaccine to determine which element of the immune response best predicts protection from subsequent SARS-CoV-2 infection. We found that higher memory B-cell frequencies, but not circulating antibody titres or T-cell responses, protected against SARS-CoV-2 infection, an effect that persisted up to 10 months after vaccination. Additionally, the third-dose SARS-CoV-2 vaccine led to a significant increase in both memory B-cell frequencies and circulating antibody titres, but not T-cell responses, and these increases in immune response also persisted at 10-month follow-up. These data clearly establish SARS-CoV-2–specific memory B-cell frequencies as an important correlate of protection from future SARS-CoV-2 infection.

Both SARS-CoV-2–specific plasma cells and memory B cells significantly increased after administration of the third-dose of SARS-CoV-2 vaccine. Significantly higher memory B-cell frequencies were observed in those who did not develop incident infection, when measured both prior to and 14 days after the third-dose SARS-CoV-2 vaccination. Furthermore, higher day 14 S-specific memory B-cell frequencies were associated with a reduced risk of infection up to 10 months after vaccination. This suggests that higher memory B-cell frequencies serve as reliable correlate of protection from SARS-CoV-2 infection. The significant increase in memory B-cell frequencies induced by the third-dose vaccination is consistent with previous studies (2, 3), and lower memory B-cell frequencies have previously been reported in cases of breakthrough SARS-CoV-2 infection with the Delta variant in one cohort study of close contacts when measured at the time of exposure (6). Our prospective study, conducted during the emergence of the OM variant and over a longer follow-up period (median of 90 days between vaccination and infection), builds on the evidence supporting memory B-cell frequencies as an important correlate of protection from infection.

Despite antibody titres also significantly increasing after the third-dose vaccine, we found no association between either pre- or post-booster vaccination binding antibody titres and protection from infection in our analyses. SARS-CoV-2 mRNA-based vaccination has been demonstrated to induce a persistent GC B-cell response, with SHM frequencies of S-specific GC B cells increasing over time, resulting in S-specific memory B cells with high levels of SHM (4). We speculate that, although higher binding antibody titres offer protection against SARS-CoV-2 infection, the relative protection offered by memory B-cell frequencies may increase over time since vaccination as circulating antibody levels decline and SHM accumulates. This is in keeping with our findings, where despite the significant rise in antibody titres in response to vaccination, higher antibody titres were not associated with protection from subsequent infections.

We did not observe significant changes in memory T-cell responses following the third-dose vaccination. SARS-CoV-2 mRNA vaccination has previously been shown to induce S-specific CD4^+^ T cells with a gradual and more variable development of CD8^+^ T-cell responses (23), which differentiate into memory cells (16). Typically, it takes several weeks after immunisation for memory T cells with a high proliferative capacity to develop (24), which may explain the absence of a significant increase at 14 days after the third-dose vaccination.

The duration of effective immunity after SARS-CoV-2 vaccination remains controversial. Although circulating antibody titres decreased at 10 months after vaccination, they remained significantly higher than that before the third-dose titres, consistent with previous reports (25). Long-lasting memory B-cell responses have also previously been reported in convalescent COVID-19 (26). That memory B-cell responses persisted to 10 months in our study suggests that vaccines may provide a longer duration of protection against SARS-CoV-2 infection than what is measured through measurement of circulating antibody titres alone.

Memory B cells may serve as a promising correlate of protection in responses to several viral vaccines. The emergence of GC memory B-cell responses has been previously demonstrated following influenza vaccination (27) and may play a pivotal role in broadening the spectrum of vaccine-induced protective antibodies against mutating viral pathogens such as SARS-CoV-2 or influenza. SARS-CoV-2 vaccine mRNA and S antigen has been detected in the axillary lymph nodes of vaccinated but only rare foci of S antigen in the lymph nodes of previously infected individuals for up to 2 months following vaccination (28). This ongoing antigen presentation following mRNA vaccination may broaden the immune response to viral variants by stimulating B cells with lower affinity for the original S epitopes, potentially leading to increased binding to variant epitopes. This appears to be unique to SARS-CoV-2 mRNA vaccination as such long-lasting GC reactions have not been observed after other vaccinations such as influenza (27). The role of T cells in protection from development of SARS-CoV-2 infection is unclear but may play a similar role to other viral infections such as influenza, where T cells are thought to contribute more to protect against severe disease rather than affecting the risk of acquisition of infection (29, 30).

During the follow-up period from December 2021 to April 2022 in Ireland, the dominant SARS-CoV-2 variants were OM BA.1 and BA.2 (20), which exhibited the greatest extent of immune evasion of all VOCs at that time (31). Memory B cells could provide a second layer of defence against challenge by variant pathogens, whereas T-cell responses may have a more pivotal role in the underlying control of significant tissue damage underpinning the defence against severe disease. Studies have demonstrated that the third dose of WT virus mRNA significantly enhances the production of neutralising antibodies against highly mutated strains such as OM (32, 33). Additionally, the third dose expands the generation of memory B cells targeting subdominant epitopes that are less mutated in OM, boosting anti-variant neutralising antibodies (34). The ongoing GC responses in B cells result in increased SHM within memory B cells, leading to the development of neutralising antibodies with heightened affinity (35).

Furthermore, the third SARS-CoV-2 vaccine dose has been shown to enhance the quantity and diversity of RBD-specific memory B cells, including the emergence of novel, antibody-producing clones with significantly increased potency, targeting more conserved regions of the RBD (36). Monoclonal antibodies sequenced from these cells showed increased potency and breadth against multiple variants, including OM (36). This enhanced memory B-cell response may explain the observed protection from OM infection in individuals with higher memory B-cell frequencies following a third mRNA vaccine dose in this study, despite the vaccine not being specifically tailored to SARS-CoV-2 variants.

There are limitations to this study. The population consisted of relatively young participants. Additional research involving older populations would be necessary to determine the generalisability of these findings across diverse demographic groups at higher risk of severe COVID-19. During this period, participants followed prevailing public health guidance to test if symptomatic or if a close contact, which may not have captured all incident infections, particularly asymptomatic infections, even though a number of asymptomatic infections were indeed reported. As participants self-reported incident SARS-CoV-2 infection, we were unable to collect more detailed specimen data related to the SARS-CoV-2 pathogen, such as the VOC or the viral inoculum size. The limited sample size of the T-cell sub-study should be considered when interpreting the results, as there may have been insufficient statistical power to detect smaller changes. Furthermore, there are limitations to the scope of this study including the lack of data for assessing correlates against other outcomes apart from SARS-CoV-2 infection (e.g., severity of COVID-19 and viral shedding).

Despite these limitations, this study provides novel insights into which components of the immune response to SARS-CoV-2 vaccination predict protection against SARS-CoV-2 infection. To the best of our knowledge, this is the first multi-parameter study to describe which components of the immunological response to SARS-CoV-2 vaccination predict protection from SARS-CoV-2 infection. We demonstrated that higher memory B-cell frequencies, rather than circulating antibody titres or T cells before and after the third-dose vaccination, best predicted protection from incident SARS-CoV-2 infection. Further research with longer follow-up time is needed to establish the durability of this protection. However, this study clearly establishes memory B cells as a correlate of protection from infection. This knowledge could identify individuals in whom to prioritise future booster vaccines and therefore help to guide targeted future vaccine schedules alongside future vaccine efficacy studies with implications beyond SARS-CoV-2 to other viral pathogens.

## Data Availability

The raw data supporting the conclusions of this article will be made available by the authors, without undue reservation.

## Group members of The All Ireland Infectious Diseases Cohort Study

Mater Misericordiae University Hospital: A. Cotter, E. Muldoon, G. Sheehan, T. McGinty, J. S. Lambert, T. O’Gorman, C. Kelly, and K. Leamy. St Vincent’s University Hospital: J. Byrne, G. Kenny, K. McCann, R. McCann, C. O’Broin, S. Savinelli, J. O’Halloran, E. Feeney, and P. W. G. Mallon. CEPHR: A. Garcia Leon, S. Miles, D. Alalwan, R. Negi, G. Saini, and E. Moore. Beaumont Hospital: E. de Barra, S. McConkey, and K. Hurley, B. Jacob. St. James’ Hospital: F. Lyons. University College Cork: M. Horgan and C. Sadlier. University College Dublin: T. Bracken. Sligo University Hospital: B. Whelan. Our Lady of Lourdes Hospital: J Low. Wexford General Hospital: O Yousif. University Hospital Galway: B. McNicholas. St. Luke’s Hospital Kilkenny: G. Courtney. Children’s Health Ireland: C. O’Maoldomhnaigh and P. Gavin.

## Group members of VACCELERATE - EU-COVAT-1 Part A Study Group

University of Cologne: Julia M. Neuhann, Jannik Stemler, Ullrich Bethe, Sarah Heringer. Jon Salmanton-García, Lea Tischmann, Arnd Cüppers, Jan Grothe, Philipp Koehler, and Oliver A. Cornely. Hospital La Paz: Antonio J. Carcas and Jesús Frías-Iniesta. Hacettepe University School of Medicine: Murat Akova. CEPHR: Alejandro Garcia Leon, Patrick Mallon, Riya Negi, Colette Gaillard, and Gurvin Saini. University of Antwerp: Christine Lammens, An Hotterbeekx, Katherine Loens, Surbhi Malhotra-Kumar, Herman Goossens, and Samir Kumar-Singh. Medical University of Vienna: Franz König, Lusine Yeghiazaryan, and Martin Posch.
