# Supplementary material for: Robust and persistent B-cell responses following SARS-CoV-2 vaccine determine protection from SARS-CoV-2 infection
Source: Front Immunol. 2024 Sep 17;15:1445653. doi: 10.3389/fimmu.2024.1445653 (PMC11442242; doi:10.3389/fimmu.2024.1445653)
Supplement: Supplementary file 1 [file DataSheet1.docx]

**Supplementary Material:**

**Methods:**

**Serological measurement of SARS-CoV-2 specific antibodies**

We used electrochemiluminescence assays to quantify antibodies to SARS-CoV-2 spike subunit 1 (S1), spike subunit 2 (S2) and receptor-binding domain (RBD) in plasma (reported in WHO IU/mL). IgG against RBD, S1, S2 proteins were measured using the CEPHR COVID19 serologic assay, described in detail elsewhere(1). Briefly, SARS-CoV-2 RBD, S1 and S2 (Sino Biological, Inc), diluted in ChonBlock ELISA buffer (CB) (Chondrex Inc, Redmond, WA, USA), were coupled with individual MSD “linkers” Meso Scale Diagnostics, LLC (MSD, Rockville, MD). Linkers were combined to make a coating solution and added to MSD U-PLEX plates and incubated for one hour. Plates were washed in phosphate buffered saline (PBS) and 0.05% tween (Bio Sciences Ltd, Ireland). Serial dilutions (1:2) of antibodies (Sino biological, Inc) in CB were used to make a 7-point standard curve. Plasma diluted in CB and standard were added to the wells, plates were incubated for 30 minutes then washed. MSD SULFO-TAG-labelled goat anti-human IgG secondary antibody was added to the wells, incubated for one hour, washed and MSD GOLD read buffer B added. Plates were analysed with a MESO QuickPlex SQ 120 instrument (MSD, Rockville, MD, USA). The operational performance and harmonisation of the assay output to the WHO international standard for anti-SARS-CoV-2 immunoglobulin NIBSC code 20/136, Hertfordshire, UK, reported as international units (BAU)/ml) has been outlined elsewhere.(1)

**Plasma cell and memory B cell profiling with B cell ELISpot**

We assessed SARS-CoV-2 specific plasma cell and memory B cell frequencies from PBMCs using a B cell ELISpot assay. Our ELISpot protocol is based on established and standard assays in the field for detecting IgG vaccine-induced B cell responses (2-4) and adapted to detect SARS-CoV-2 Spike (S) and RBD specific frequencies. ELISpot Multiscreen filtration plates (MSIPS4W10, MilliporeSigma) were coated with 10μg/ml recombinant SARS-CoV-2 RBD protein (40592-V08H, Sino Biological), Full Spike protein(40589-V08B1, Sino Biological) or 10 μg/ml anti-human IgG (3580-2A Mabtech) overnight or up to 96 hours at 4°C. The anti-human IgG-coated wells serve as controls to measure total IgG-secreting cells and allow us to determine the proportion of SARS-CoV-2-specific antibody-secreting cells (ASC) within the total population of ASCs. Plates were washed five times with dPBS and blocked with complete RPMI (cRPMI, RPMI 1640 Medium, GlutaMAX™ supplemented with 50μg/ml Penicillin–Streptomycin and 10%FCS (10500-064,Gibco))for one hour at room temperature (RT).

Cryopreserved PBMCs were thawed and allowed to rest overnight for plasma cell measurement or stimulated ex-vivo with 1μg/ml R848 (3580-2A , Mabtech) and 10ng/ml recombinant human (rh)IL-2 (3580-2A, Mabtech) for 5 days at 37°C, 5%CO2 for memory B cells to differentiate ex-vivo into ASCs.Cells were washed to remove secreted antibodies. 500,000 cells per well (antigen specific coated wells) or 50,000 cells per well (anti-human IgG coated wells) were added in cRPMI to the plates in duplicates and incubated for 18 hours at 37°C, 5%CO2. Next day, cells were discarded and plates were washed with dPBS and incubated with Biotinylated anti-Human IgG (1μg/ml, 3580-2A, Mabtech ) for two hours at RT then incubated with Streptavidin-Alkaline-Phosphatase (SA–ALP) (3580-2A, Mabtech ) for one hour at RT followed by BCIP/NBT-plus substrate (3650-10, Mabtech) and incubated at RT until spots were clearly observed. Plates were analysed using an ELISpot Reader Astor (Mabtech).

For the plasma cells, Antigen-specific IgG-Secreting B Cells were expressed as spot forming unit (SFU) per 10^6 PBMCs. For the memory B cells, antigen-specific IgG-secreting B cells were expressed as SFU per total IgG-secreting B cells (IgG+ ASC) controls. This approach allows us to determine the proportion of SARS-CoV-2-specific ASCs within the total population of ASCs. The analyser was blinded to the infection outcome.

**SARS-CoV-2-Specific memory T cell ELISpot assay**

Human IFN-γ (ALP) Plates (3420-4APW, Mabtech) were washed five times with dPBS and blocked with complete RPMI (cRPMI, RPMI 1640 Medium, GlutaMAX™ supplemented with 50 μg/ml Penicillin–Streptomycin and 10%FCS (10500-064,Gibco)) for one hour at 37°C. Cryopreserved PBMCs were thawed and allowed to rest two hours at 37°C, 5%CO2 before seeding. Cells were washed twice with complete RPMI. 500,000 cells per well were added in 100μl of cRPMI with 2μg /ml of SARS-CoV-2 WT S peptide (PepTivator SARS-CoV-2 Prot_S Complete, Miltenyi), SARS-CoV-2 WT N peptide (PepTivator SARS-CoV-2 Prot_N, Miltenyi), SARS-CoV-2 Omicron S BA4&BA5 peptide (PM-SARS-SMUT10, JPT) and SARS-CoV-2 Omicron N peptide (PepTivator SARS-CoV-2 Prot_N B.1.1.7, Miltenyi) to the plates in duplicates  and incubated for 18 hours at 37°C, 5%CO2. DMSO and 1μg/ml of anti-CD3 (3420-4APW, Mabtech) were added as negative and positive control for each sample. Next day, cells were discarded, and plates were washed with dPBS and incubated with Biotinylated anti-IFN γ (3420-4APW, Mabtech) for 2 hours at room temperature (RT) then incubated with Streptavidin-Alkaline-Phosphatase (SA–ALP) (3420-4APW, Mabtech) for 1 hour at RT followed by BCIP/NBT-plus substrate (3420-4APW, Mabtech) and incubated at RT until spots were clearly observed. Plates were analysed using an ELISpot Reader Astor (Mabtech). The SARS-CoV-2 specific memory T cells were expressed as spot forming unit (SFU) per 0.5X10^6^ PBMCs subtracted with negative control.

**References**

1. Kenny G, Negi R, O'Reilly S, Garcia-Leon A, Alalwan D, Gaillard CM, et al. Performance and validation of an adaptable multiplex assay for detection of serologic response to SARS-CoV-2 infection or vaccination. Journal of Immunological Methods. 2022;510:113345.

2. Jahnmatz M, Kesa G, Netterlid E, Buisman AM, Thorstensson R, Ahlborg N. Optimization of a human IgG B-cell ELISpot assay for the analysis of vaccine-induced B-cell responses. J Immunol Methods. 2013;391(1-2):50-9.

3. Rouers A, Tay MZ, Ng LFP, Renia L. B-cell ELISpot assay to analyze human memory B cell and plasmablast responses specific to SARS-CoV-2 receptor-binding domain. STAR Protoc. 2023;4(1):102130.

4. Tay MZ, Rouers A, Fong SW, Goh YS, Chan YH, Chang ZW, et al. Decreased memory B cell frequencies in COVID-19 delta variant vaccine breakthrough infection. EMBO Mol Med. 2022;14(3):e15227.
